# Discovery of IL-8/CXCL8 (The Story from Frederick)

**DOI:** 10.3389/fimmu.2015.00278

**Published:** 2015-06-05

**Authors:** Teizo Yoshimura

**Affiliations:** ^1^Laboratory of Molecular Immunoregulation, Cancer and Inflammation Program, Center for Cancer Research, National Cancer Institute, Frederick, MD, USA

**Keywords:** chemokines, chemoattractants, IL-8, MCP-1, inflammation

The infiltration of leukocytes is a specific, rather than a random, event, and regulated by the production of chemoattractants that specifically attract a certain type of leukocytes. By early 1980s, chemoattractants, now referred as “classical chemoattractants,” including the bacterial peptide *N*-formyl-methionyl-leucyl-phenylalanine (FMLF), the C5a fragment of serum complement and the lipid mediator leukotriene B4 (LTB4), had been identified. However, FMLF and C5a lacked cell specificity and attracted both neutrophils and monocytes. LTB4 exhibited both chemotactic and chemokinetic activities for phagocytes. There were several reports suggesting the existence of other cytokine-like chemoattractants. Lymphocyte-derived chemotactic factor (LDCF) was detected in the culture supernatant of mitogen-activated peripheral blood mononuclear cells (PBMC) (1). A neutrophil chemoattractant was also present in the culture supernatant of activated monocytes or macrophages (2). However, these cytokine-like chemoattractants were not purified or cloned.

The early 80s was the time when various cytokines were finally purified and/or cloned by immunologists owing to the technological advance in protein purification and molecular cloning. The laboratory of Joost J. Oppenheim was focused on investigations of interleukin 1 (IL-1) and established that it was produced by many cell types and had multiple biological activities by also stimulating a great variety of cell types. Subcutaneous injections of IL-1 had been shown to induce acute inflammatory responses with rapid margination of neutrophils followed by their extravascular infiltration (3). They therefore tested the chemotactic effects of partially purified human epithelial cell-derived thymocyte-activating factor (ETAF); an epithelial cell-derived IL-1. ETAF purified by elution from Sephadex gels was found to attract both polymorphonuclear and mononuclear leukocytes *in vitro* (4). Since ETAF was biochemically identical to IL-1, it was concluded that IL-1 was chemotactic. These findings were confirmed by Dinarello and his colleagues (5). In addition to IL-1, recombinant human TNF was shown to be chemotactic for neutrophils by another group (6). These findings suggested that cytokine-like chemoattractants produced by activated mononuclear leukocytes reported earlier could be attributed to IL-1 and/or TNF. However, the Oppenheim laboratory was concerned about the fact that their preparations of ETAF/IL-1were not pure and pursued opportunities to investigate more purified preparations of IL-1 (personal communication with Joost J. Oppenheim).

I studied inflammation in Hideo Hayashi’s laboratory in Kumamoto, Japan from 1979 to 1985. My research was focused on three putative macrophage chemoattractants partially purified from skin extracts of guinea pig delayed hypersensitivity reaction, one of which was thought to be the guinea pig version of LDCF. Unfortunately, it was never purified to determine its identity. I met Ed Leonard in 1984 when he came to Japan to attend The 10th International Reticuloendothelial System Congress. His lab was interested in identifying the nature of chemoattractants derived from activated mononuclear leukocytes or bacteria. He expressed his interest in our guinea pig chemoattractants and recruited me to join his laboratory. In 1985, I completed my PhD project and came to Ed’s lab on a fellowship supported by the Japanese Foundation for Promotion of Cancer Research. I brought partially purified guinea pig LDCF to study, but that plan was immediately scratched when Ed and Tibor Borsos, chief of our lab, found out that guinea pig skin was treated with acetone before protein extraction. They were concerned about possible artifacts due to acetone treatment.

We were very much experienced in evaluating the activity of chemoattractants by examining their potency and efficacy using the 48-well multi-well chemotaxis chambers developed by Ed (7) (Figure 1). In 1986, I published my first paper in Ed’s lab examining the oxidation of FMLF by neutrophils and I was ready for a larger task. Since we were very intrigued to learn that the cytokine IL-1 was chemotactic for neutrophils and monocytes, we decided to examine the chemotactic activity of IL-1 in more detail. In collaboration with the Oppenheim lab, we examined the chemotactic activity of highly purified native IL-1 or recombinant IL-1. To our surprise, neither of them was chemotactic for neutrophils or monocytes. We speculated that IL-1 might indirectly attract neutrophils by inducing the release of a chemoattractant such as LTB4 by neutrophils. We tested this hypothesis, but there was no chemotactic activity in the supernatant of IL-1-stimulated neutrophils. We also examined whether IL-1 could augment neutrophil migration induced by other chemoattractants, such as FMLF, or activate basophils to release neutrophil chemoattractants. Again, IL-1 showed no effects. These results led us to conclude that the partially purified IL-1 preparation was contaminated by a neutrophil chemoattractant, which was different from IL-1. The supernatant of LPS-activated PBMC or monocytes, which are rich sources of IL-1 and used as a source of partially purified IL-1, indeed also contained a potent neutrophil chemotactic activity, further supporting our conclusion (8).

**Figure 1:**
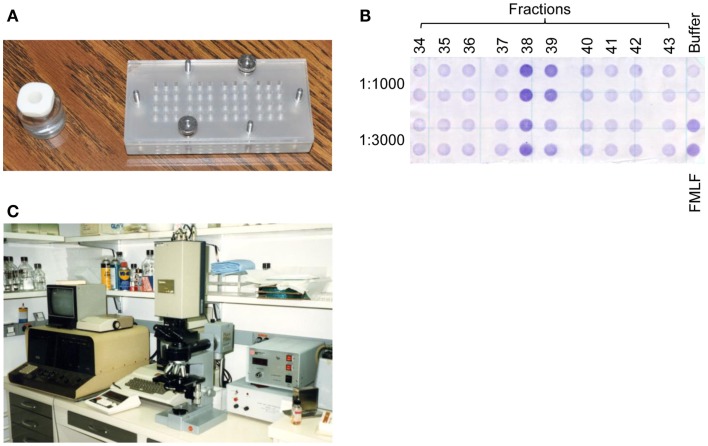
**A chemotaxis assay system used to purify MDNCF**. **(A)** Previously used individual chemotaxis chamber (left) vs. 48-multi-well chemotaxis chamber (right). **(B)** Consecutive fractions eluted from a reversed-phase HPLC column were assayed in duplicate at two different dilutions (1:1000 and 1:3000) in a single chamber with a negative (buffer) and positive (FMLF) control, and migrated cells on the bottom of the filter were stained (from the original notebook). The fraction 38 had the peak activity. **(C)** The percentage of migrated cells was analyzed using an image analyzer.

Kouji Matsushima, head of a group in the Oppenheim lab, had a vast knowledge and experience in protein purification and had just succeeded in purifying IL-1. Because the Leonard and the Oppenheim lab were both interested in identifying the protein, which contaminated crude IL-1 preparations, Kouji and I quickly established collaboration and decided to biochemically identify this activity. Since we were both from Japan, we had no language barrier. We first isolated neutrophil chemotactic activity from IL-1 activity contained in the culture supernatant of LPS-activated PBMC (8), and then achieved the first purification by the end of 1986. This protein was the first chemoattractant with target cell specificity and we termed the protein monocyte-derived neutrophil chemotactic factor (MDNCF) based on its activity and cellular source (9).

The N-terminal amino acid sequence of MDNCF was determined in Ettore Appella’s laboratory, NCI. It was realized that MDNCF exhibited considerable amino acid sequence similarity at the N-terminus to β-thromboglobulin (β-TG), platelet factor 4 (PF4), and γIP-10. The β-TG precursor CTAPIII (connective tissue activating protein) stimulated replication of connective tissue cells, suggesting its role in wound healing. PF4 was a chemoattractant. γIP-10 was an interferon-induced product of the U937 monocyte-like cell line. Thus, MDNCF not only structurally but also functionally belonged to this family of small proteins involved in inflammation (9). β-TG, PF4, and γIP-10 were subsequently determined as the members of the chemokine family (10). When we submitted our manuscript to a journal for publication, we had considerable difficulty overcoming the criticisms of one of the reviewers, who maintained that detection of a cell-derived chemoattractant for neutrophils was redundant with already well known chemotactic factors such as FMLF and C5a, and was therefore not of any importance. It has recently become clear that a signal relay of multiple chemoattractants is critical for the precise trafficking of leukocytes to sites of tissue injury (11); thus, chemoattractants have a non-redundant role.

Shortly after the publication of our report in 1987, the laboratories of Marco Baggiolini and Jo Van Damme also reported the purification of the identical protein (12, 13). At a meeting in Baden, Austria, it was agreed by the co-discoverers of this cell-derived neutrophil chemoattractant to name this activity neutrophil-activating peptide (NAP-1). However, Larsen and coworkers in the Oppenheim’s laboratory subsequently discovered that NAP-1 also chemoattracted a subset of T lymphocytes and they therefore proposed to rename it as interleukin-8 (IL-8) (14). Although this proposal was eventually accepted, it was disliked by the interleukin aficionados because they did not consider chemotactic effects very important (personal communication with Joost J. Oppenheim).

During the purification of MDNCF, we noticed that a monocyte chemoattractant was also present in the culture supernatant of LPS-activated PBMC. However, purification of the monocyte chemoattractant was more difficult because the amount of this chemoattractant in the supernatant was lower than that of MDNCF. Subsequently, both the Leonard and the Oppenheim laboratories independently purified the protein, monocyte chemoattractant proten-1 (MCP-1) or macrophage chemotactic and activating factor (MCAF), respectively, from the culture supernatant of tumor cell lines and published a paper in 1989 (15, 16). We believed that MCP-1 was identical to LDCF. Unexpectedly, amino acid sequence analysis of this MCP-1 revealed the presence of four half cysteine residues at almost identical locations to those of MDNCF, although the overall amino acid sequences between the two proteins were not highly conserved. There was no extra amino acid between the first two half cysteine residues in MCP-1 (CC vs. CXC). By this time, there were reports of additional proteins belonging to this protein family, including RANTES, pLD78, TCA3, MIP, and JE. Although these proteins were later determined as chemokines (JE turned out to be the mouse ortholog of human MCP-1) (10), MCP-1 was the only protein identified based on its biological capacity to selectively attract monocytes. Identification of IL-8 and MCP-1 thus pointed out the existence of a family of chemotactic cytokines with leukocyte specificity and led to the identification of the protein family chemokines with at least two CXC and CC subgroups. This family was subsequently referred to as chemokines, an abbreviated version of “chemoattractant cytokines,” by Oppenheim.

As noted above, we began our study by asking a simple question whether IL-1 was indeed a chemotactic factor. The results of the study led us to the discovery of IL-8, the first cytokine-like chemoattractant with cell specificity, and most importantly, which in turn led to the discovery of a novel protein family with chemotactic activity. This was an example of a successful collaboration between two laboratories with complementary strength. We were extremely fortunate to be the first to identify IL-8 and MCP-1 as evidenced by being awarded the patents for each, because the field was ripe for the discovery and many laboratories were just behind us.

## Conflict of Interest Statement

The author declares that the research was conducted in the absence of any commercial or financial relationships that could be construed as a potential conflict of interest.
